# Ion-Imprinted Polymer Structurally Preorganized Using a Phenanthroline-Divinylbenzoate Complex with the Cu(II) Ion as Template and Some Adsorption Results

**DOI:** 10.3390/polym15051186

**Published:** 2023-02-26

**Authors:** Egla Yareth Bivián-Castro, Abraham Zepeda-Navarro, Jorge Luis Guzmán-Mar, Marcos Flores-Alamo, Brenda Mata-Ortega

**Affiliations:** 1 Centro Universitario de los Lagos, Universidad de Guadalajara, Av. Enrique Díaz de León 1144, Col. Paseos de la Montaña, Lagos de Moreno 47460, Jalisco, Mexico; 2 Facultad de Ciencias Químicas, Universidad Autónoma de Nuevo León (UANL), Ave. Universidad s/n, Cd. Universitaria, San Nicolás de los Garza 66455, Nuevo León, Mexico; 3 Facultad de Química, Universidad Nacional Autónoma de México, Ciudad Universitaria, Ciudad de México 04510, Ciudad de México, Mexico

**Keywords:** copper(II) ion template, 4-vinylbenzoic acid, adsorption capacity, ion-imprinted polymer, heavy metals

## Abstract

The novel [Cuphen(VBA)_2_H_2_O] complex (phen: phenanthroline, VBA: vinylbenzoate) was prepared and used as a functional monomer to preorganize a new ion-imprinted polymer (IIP). By leaching the Cu(II) from the molecular imprinted polymer (MIP), [Cuphen(VBA)_2_H_2_O-*co*-EGDMA]*_n_* (EGDMA: ethylene glycol dimethacrylate), the IIP was obtained. A non-ion-imprinted polymer (NIIP) was also prepared. The crystal structure of the complex and some physicochemical, spectrophotometric techniques were also used for the MIP, IIP, and NIIP characterization. The results showed that the materials are nonsoluble in water and polar solvents, which are the main features of polymers. The surface area of the IIP is higher than the NIIP demonstrated by the blue methylene method. The SEM images show monoliths and particles smoothly packed together on spherical and prismatic-spherical surfaces in the morphology of MIP and IIP, respectively. Moreover, the MIP and IIP could be considered as mesoporous and microporous materials, shown by the size of the pores determined by the BET and BJH methods. Furthermore, the adsorption performance of the IIP was studied using copper(II) as a contaminant heavy metal. The maximum adsorption capacity of IIP was 287.45 mg/g at 1600 mg/L Cu^2+^ ions with 0.1 g of IIP at room temperature. The Freundlich model was found to best describe the equilibrium isotherm of the adsorption process. The competitive results indicate that the stability of the Cu-IIP complex is higher than the Ni-IIP complex with a selectivity coefficient of 1.61.

## 1. Introduction

Heavy metals represent a critical contamination in bodies of water around the world; they are persistent pollutants that can never be destroyed, and they tend to bio-accumulate in living organisms. Nickel (Ni), copper (Cu), manganese (Mn), cadmium (Cd), iron (Fe), cobalt (Co), zinc (Zn), arsenic (As), chromium (Cr), mercury (Hg), and lead (Pb) are some examples of metals belonging to this classification; these metals cause an environmental impact due to their toxicity, and they have physicochemical characteristics such as high density, mass, and atomic weight above 20. The contribution of these metals to the hydrological cycle comes from various sources, one of them being lithogenic or geochemical in origin from minerals due to erosion, rain, etc. In addition, industry, economy, domestic waste, etc. are some examples of anthropogenic sources of heavy metals with an important environmental impact [1,2]. Although metals like copper are essential for all living organisms as trace dietary minerals and their deficiency alters the normal functions of the human body, causing several diseases, they can be toxic at higher or even at low levels, thus posing health risks. In nature, copper comes from minerals and it is an essential trace element for the healthy function of the living organisms, because it is part important of metalloen-zymes as their active center. Copper can coordinate with various ligands of oxygen (O), nitrogen (N), and sulfur (S) donor atoms, forming a variety of geometric structures such as a flat square, a square pyramidal, a trigonal bipyramidal, or an octahedral. Then, taking advantage of the characteristics mentioned, copper has been used as a template to preorganize several desirable structures with the objective to design materials inspired by nature with specific cavities. These new materials have been applied to selectively remove heavy metals with chemical characteristics, with radii and charge coming from the metal ion templates used [3,4]. Throughout the world, different analyses have been carried out, referring to the evaluation of the levels of heavy metals in surface waters. During the lockdown period of COVID-19 (coronavirus pandemic 2019), the levels of pollution diminished, representing an opportunity to improve the quality of natural resources [5]. Hence, several methods were applied for the extraction of contaminated metal ions. An accessible methodology to purify water is adsorptive separation by substances with a significant active increase in surface area, chemical and thermal stability, a variety of functional groups, and significant adsorption efficiency and efficacy [6,7]. A search for effective adsorbents to remove heavy metals is a popular endeavor of the scientific community around the world. Some previous reports suggested adsorbents of natural (e.g., biomass, minerals, agriculture, and animal waste) [8,9] and synthetic (e.g., inorganic polymers, chelating resins, and cross-linked polymers) [7,10] origin. Recently, ion-imprinted polymers (IIPs) have been used in water treatment for the removal and recovery of heavy metals due to their high selectivity, being optimal candidates for this type of treatment. Imprinted polymers technology is based on the elaboration of highly stable synthetic polymers called molecular imprinted polymers (MIPs). These have selective molecular recognition characteristics because there are locations within the polymer matrix that are complementary to the analyte in terms of functional group shape and position, which are able to identify the template molecule and are based on the functioning models of biological systems [11,12]. In this paper, a novel adsorbent, with the characteristics mentioned above of IIP, was prepared. This new material has specific cavities to catch heavy metals from contaminated waters. The chemical structure of the IIP was carefully built step by step. Firstly, for the first time, a copper complex of [Cuphen(VBA)_2_H_2_O] (phen: phenanthroline, VBA: vinylbenzoate) was prepared; its structure was completely elucidated by X-ray diffraction results using the corresponding blue crystals. The copper complex had a double purpose: 1. as the functional monomer and 2. as the ion template, the Cu(II). A radical polymerization reaction was found using EGDMA (ethylene glycol dimethacrylate) as the cross-linker agent and dimethylformamide as the porogen. The corresponding MIP of [Cuphen(VBA)_2_H_2_O-*co*-EGDMA]*_n_* was obtained as a green crystalline material. Finally, after a soft acidic leaching of the Cu(II) from the MIP, the IIP was obtained as pale yellow crystals. In a similar way, with the exception of the copper complex step, the non-ion-imprinted polymer (NIIP) was prepared. Physicochemical tests and spectrophotometric techniques were used to study the chemical structure of the materials. In addition, the surface morphology of the materials was analyzed, and some adsorption experiments were performed, suggesting the capacity, the selectivity, and the method for heavy metal adsorption by the IIP.

## 2. Materials and Methods

Preparation and characterization of Cu(II)-phenanthroline vinylbenzoate complex and the corresponding MIP, IIP, and NIIP.

### 2.1. Reagents

The 4-Vinylbenzoic acid (VBA), ethylene glycol dimethacrylate (EGDMA), azobiisobutyronitrile (AIBN), and 1,10-phenanthroline (phen) were provided by Sigma-Aldrich (Estado de México, México). All other reagents were of AR grade.

### 2.2. Apparatus

A freshly methanolic solution of 0.001 M was prepared for conductivity measurements; a Conductronic PC45 electrode (Conductronic, Puebla, México) was used. A Sherwood Scientific LTD magnetic balance Magaway MSB Mk1 (Sherwood Scientific, Cambridge, United Kindom) model was used for the magnetic susceptibility determinations. Pascal’s constants were used for the diamagnetism corrections [13]. FT-IR spectra were recorded in the frequency range 4000–400 cm^−1^ by the KBr pellet method using Perkin Elmer Spectrum RXI (Perkin Elmer, Waltham, MA, USA). Elemental analyses (C, H, N) were performed at ALS Environmental‘s Tucson Laboratory (ALS Environmental, Tucson, AZ, USA). N_2_-physisorption analysis of materials was performed on previously out-gassed samples at 120 °C using a Micromeritics Instrument Corporation model, Tristar II Plus (Micromeritics Instrument Co., Norcross, GA, USA). The Brunauer-Emmett-Teller (BET) technique was used to calculate the specific surface area, and the Barret-Joyner-Halenda (BJH) method was used to calculate the pore volume. Scanning electron microscopy (SEM) was used to identify morphology and particle size, using a JEOL model, JSM-6490LV (JEOL Ltd., Tokyo, Japan), at 20 kV.

### 2.3. Synthesis of [Cuphen(VBA)_2_H_2_O] Functional Monomer

**Scheme 1:**
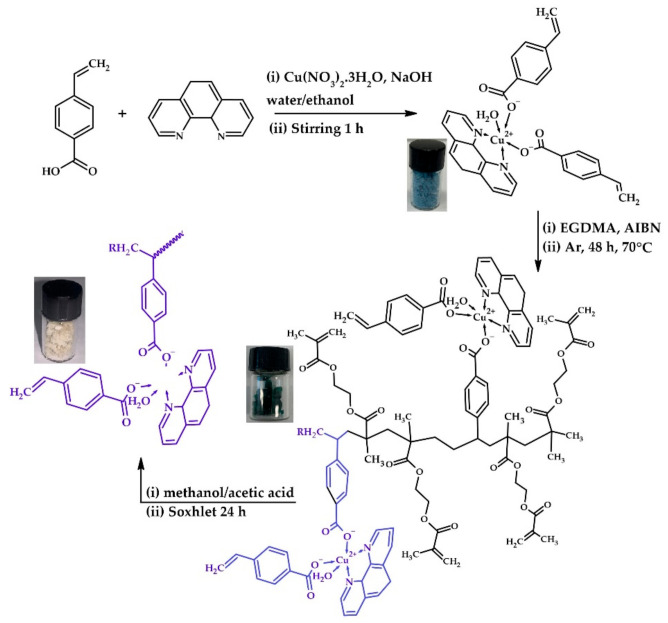
General steps of the preparation of the materials: the functional monomer, the MIP, and the IIP.

In 20 mL of deionized water, 1 mmol (0.148 g) of vinylbenzoic acid and 1 mmol of sodium hydroxide were completely mixed; then, 1 mmol of Cu(NO_3_)_2_·3H_2_O previously dissolved in 5 mL of deionized water was added. Finally, the ethanolic solution of 1 mmol of phenanthroline (0.180 g) in 10 mL of ethanol was added, and the reaction was left under a stirrer for 1 h at room temperature. The resulting blue precipitate was filtered off and then dissolved in 15 mL of methanol and maintain in the refrigerator to obtain crystals available for X-ray diffraction. The results were as follows: [Cuphen(VBA)_2_H_2_O] Λ_M_ 73 cm^2^/Ω mol, *χ_g_* = 3.2 × 10^−6^ cm^3^/g, M.B. = 2.0. El. Anal. Exp. C 60.31, H 4.53, N 5.82% Calc. C 57.01, H 4.78, N 6.43%. The general steps of the functional monomer preparation are represented in Scheme 1.

### 2.4. Preparation of [Cuphen(VBA)_2_H_2_O-co-EGDMA]_n_ (MIP), the NIIP, and [Phen (VBA)_2_H_2_O-co-EGDMA]_n_ (IIP)

Ten mL of dimethylformamide (DMF) was dissolved in 0.18 mmol (0.1 g) of the copper complex, [Cuphen(VBA)_2_H_2_O]. Then, 3.59 mmol (678 μL) of EGDMA and 0.06 mmol (0.0099 g) of AIBN previously recrystallized in methanol were added. The reaction was left under constant stirring for 48 h at 70 °C and under inert atmospheric conditions. A dark green gel formed; and after being washed with deionized water, the MIP was obtained as a green solid with crystalline morphology. When nitromethane is the porogen instead of DMF and the amount of initiator is diminished, a brownish-green precipitate is formed (see Supplementary Material). A similar procedure was performed for the NIIP preparation: First, 0.035 mmol (0.0529 g) of VBA with 1 mmol of NaOH was dissolved in 10 mL of DMF, and 0.017 mmol (0.0324 g) of phen was added to this mixture. Then, 3.59 mmol of EGDMA and 0.06 mmol of AIBN were added. The polymerization reaction proceeded under the same conditions of the MIP. The NIIP was obtained as a light-brown crystalline solid. To obtaine the ion-imprinted polymer, the copper(II) ion template was removed by an acid wash as follows: First, 0.3 g of MIP was washed in 150 mL of methanol/acetic acid (9:1, *v/v*) in a Soxhlet apparatus for 24 h. After a deionized water wash, a pale-yellow crystalline solid of the IIP was finally recovered. To remove the excess water, the crystalline polymers were finally washed with methanol. The general steps of the preparation of the materials, the MIP and the IIP, are represented in Scheme 1.

### 2.5. Crystallography of the Functional Monomer, [Cuphen(VBA)_2_H_2_O]

A suitable crystal of copper complex [Cuphen(VBA)_2_H_2_O] was mounted onto glass fiber by using perfluoropolyether oil and cooled rapidly in a stream of cold nitrogen gas. Diffraction data were collected by using the Oxford Diffraction Gemini Atlas diffractometer (Oxford Diffraction Ltd., Abingdon, United Kingdom) at 130 K, and intensity data were collected with ω scans; these processes were conducted using the CrysAlisPro and CrysAlis RED software packages (Oxford Diffraction Ltd., Abingdon, United Kingdom) [14]. All the data were corrected by Lorentz and polarization effects, and final cell constants were determined by a global refinement; collected data were corrected for absorbance by analytical numeric absorption correction [15] using a multifaceted crystal model based on expressions from the Laue symmetry using equivalent reflections. The space group was determined based on a check of the Laue symmetry and systematic absences, and it was verified by utilizing the structure solution. The molecular structure was then solved and refined with the SHELXS-2018 [16] and SHELXL-2018 [17] programs (Institute of Inorganic Chemistry, Göttingen, Germany). All non-hydrogen atoms located in successive Fourier maps were treated as a riding model on their parent C atoms, while H atoms of the water (O—H) group were located in a difference map and refined isotropically with Uiso(H) of 1.5 Ueq for H—O. Anisotropic thermal parameters were applied for all non-H atoms, and fixed isotropic parameters were employed for H atoms. Drawing of the molecular structure was performed by utilizing ORTEP [18]. Crystal data and structure refinement and selected bond lengths (Å) and bond angles (o) for [Cuphen(VBA)_2_H_2_O] are shown in Table 1 and Table 2, respectively. Crystallographic data have been deposited at the Cambridge Crystallographic Data Center as Supplementary Material CCDC: 2223464.

### 2.6. Surface Area

The surface area of the IIP and NIIP was measured using the methylene blue absorption method [19,20]. From a stock solution of methylene blue (0.0176 g/L), a set of working standards were prepared to create a calibration curve. Each set was analyzed by UV-Vis spectrophotometry (Perkin Elmer, Waltham, MA, USA) at λ = 600 nm; 0.1 g of IIP and NIIP each were equilibrated with 25 mL of methylene blue solution, and samples of 1 mL by were analyzed by triplicate until the absorbance became constant. The amount of methylene blue adsorbed was evaluated based on its concentration before and after adsorption. The surface area of IIP and NIIP was calculated using the following equation:

$$As={\frac{GN_{AV}\;\Phi \times 10}{MM_{W}}}^{-20}$$

where *As* is the surface area in m^2^/g, *N_AV_* is Avogadro’s number (6.02 × 10^23^ mol^−1^), M is the mass of adsorbent (g), and G, *Φ*, and *M_W_* are the amount adsorbed (g), the molecular cross section (197.2 Å^2^), and the molecular weight (373.9 g/mol) of methylene blue, respectively.

### 2.7. Adsorption Capacity and Adsorption Isotherm

An amount of 0.1 g of IIP was added to a series of Cu(II) solutions of 25 mL with 400–1600 mg/L concentrations. All the solutions were left for 6 h under stirring at room temperature. Then, the polymer was filtered off, and the quantity of Cu(II) remaining was analyzed by atomic absorption spectrometry (AAS) using a SpectrAA 220FS model Varian (Agilent Technologies Co., Santa Clara, CA, USA). The amount of adsorbed copper ions per gram of IIP was evaluated as the adsorption capacity (Q), and it was calculated with the following equation:

$$Q=\frac{\left(C_{i}-C_{f}\right)V}{W}$$

where *C_i_* and *C_f_* are the initial and final concentration of Cu(II) (mg/L), V is the volume of the solution, and W is the mass of the sorbent, respectively.

The Freundlich adsorption isotherm was applied to understand the Cu^2+^ adsorption mechanism of the ion-imprinted polymer. The Freundlich model assumes a heterogeneous surface of the sorbent with multiple adsorption sites (bilayer) and adjacent interactions between adsorbate molecules. The linearized Freundlich equation is

$$LogQ=LogK+\frac{1}{n}logC_{e}$$

where *Q* is the metal mass adsorbed per mass unit of solid (mg/g), *C_e_* is the final concentration of solute in the aqueous phase at equilibrium (mg/L), *K* and *n* are constants, and the calculated values could be obtained from the adsorption isotherm [21,22].

### 2.8. Selective Recognition

Heavy metals as Ni(II) could be chosen as a competitor to compare the selectivity of an adsorbent like the ion-imprinted polymer by Cu(II). Niquel ion usually coordinates well with the diamine and carboxylic ligands, it has the same charge and similar ionic radii as copper. Then in principle the IIP could bind Ni(II) as well as Cu(II). A 25 mL of Ni(II) solution 426 mg/L was used, and once the adsorption equilibrium was reached, the concentration of the non-adsorbed ions in the liquid phase was determined by AAS. The distribution coefficient (K_d_), was calculated using the following equation:

$$K_{d}=\frac{(C_{i}-C_{f})V}{C_{f}W}$$

where *C*_i_, and *C*_f_, are the initial, and final solution concentrations, V is the volume of the solution, W is the mass of the sorbent. The selectivity coefficient (k), was calculated for the ratio of binding of the metal ion in a study related with the competitor species, and using the following equation:

$$k=\frac{K_{d\;\left[Cu\;\left(II\right)\right]}}{K_{d\;\left[Ni\;\left(II\right)\right]}}$$

where k represents the selectivity coefficient, K_d_[Cu(II)] and K_d_[Ni(II)] represent the distribution ratios of Cu(II) and Ni(II), respectively [21,23].

## 3. Results and Discussion

### 3.1. Crystal Structure of the Functional Monomer

**Figure 1:**
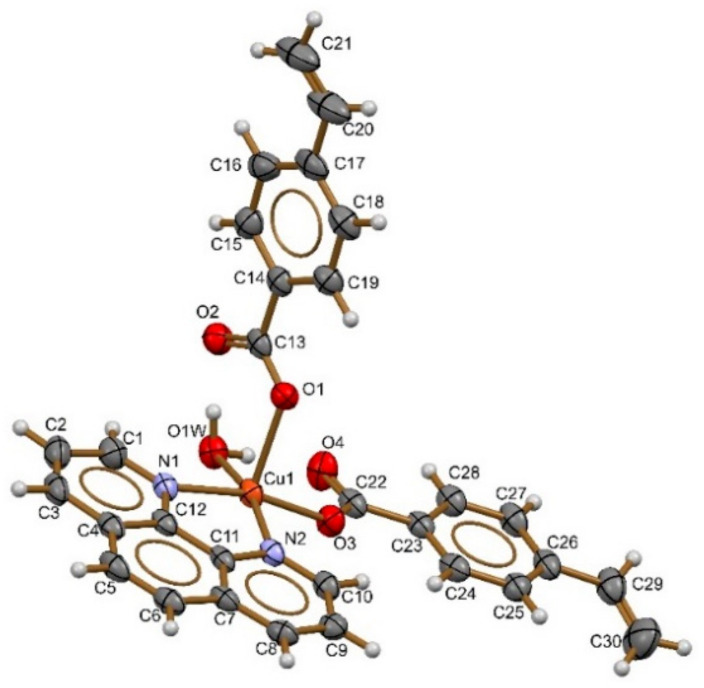
Ortep diagram of functional monomer [Cuphen(VBA)_2_H_2_O] ellipsoids at 50% of probability.

Here, the copper(II) complex was used as the functional monomer to prepare the imprinted structures. The functional monomer yields single crystals suitable for X-ray measurements. The discrete unit of coordination compound [Cuphen(VBA)_2_H_2_O] shows the coordination of the metal and atomic labeling (see Figure 1). Table 1 shows the improved cell characteristics as well as other important crystal data. In Table 2, some selected bond lengths and bond angles are shown. Copper ion has square pyramidal coordination in this molecule. The pyramid’s base is created by two Cu—N bonds made by the phenanthroline ligand’s two nitrogen atoms, and two Cu—O bonds formed by two oxygen atoms, one from the carboxylate group of the VBA ligand and one oxygen atom of the water molecule. The apical position is occupied by one oxygen atom from one of the carboxylate groups of the VBA ligand. At the base of the pyramid, the phenanthroline ligand is coplanar with the VBA ligand, with 1° deviation with a defined mean square plane 6.734 (4) x − 0.74 (10) y − 9.413 (9) z = 1.522 (3).

The major deviations of the atoms are 0.0213 Å, whereas the O(3) and the O(1W), which are also forming part of the pyramid base, are about 0.0877(2) and 0.0517(2) Å above the plane. At the apical position of the pyramid, a second vinylbenzoate anion is coordinated, with Cu—O(1) = 2.2623(16) Å, a distance similar to that observed in related compounds of 2.2623(16) [24].

The angle formed between the mean plane of the pyramid base and the apical ligand is 88.35(12)°, with a τ value of 0.025, very close to a square pyramid geometry [25]. The C(22)—O(3) coordinated bond length is longer than the C(22)—O(4) uncoordinated bond length, which is consistent with the creation of a bond between the anionic carboxylate oxygen atom and copper cation. However, a slight difference between C(13)—O(1) and C(13)—O(2) bond lengths is found due to the apically elongated square-pyramidal coordination. It is important to notice that in the complex, the C(20) and C(29) of the vinyl group are almost coplanar to the phenyl ring of the vinylbenzoate ligands. The vinyl group of the apical ligand presents some disorder, and the C(20)—C(21) and C(20A)—C(21A) bond length average corresponds to the well-established vinyl group of C(29)—C(30) = 1.302 Å bond length from the equatorial vinylbenzoate ligand [26].

**Figure 2:**
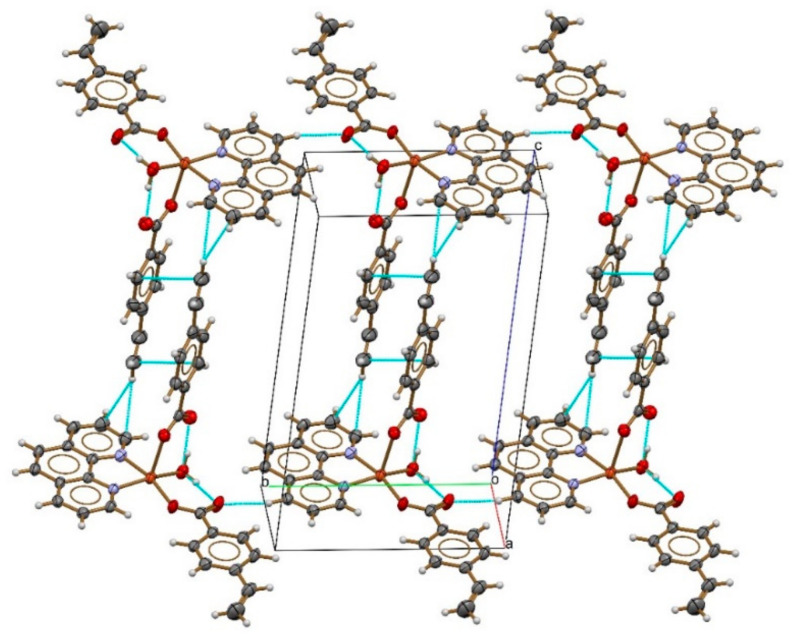
Crystal array of the functional monomer [Cuphen(VBA)_2_H_2_O], with a view along the *a* axis from the perspective of the plane formed by the *b-c* axes, emphasizing the H-bond and σ−π interactions.

In the discrete unit of [Cuphen(VBA)_2_H_2_O], the water molecule coordinated to the metal center shows two intramolecular interactions of the hydrogen bond, O(1W)—H(1WA)…O(2) and O(1W)—H(1WB)…O(4), forming a *S^1^_1_(6)* motif; in the crystal array, there are two types of intermolecular interactions—one type is hydrogen bond C—H…O, and the other is the π−π intermolecular contact (see Figure 2).

The intermolecular contact C(8)-H(8)…O(4) at 2.36 Å forms the *S^1^_1_(8)* motif along the *b* axis. Finally, the intermolecular contact of type π−π is shown between the phenyl ring C23/C27 of vinylbenzoate and N1—C1/C4—C12 of phenanthroline ligands along the plane formed by the *a-c* axes. All these interactions show a complex growing along the *b-c* plane.

### 3.2. Synthesis and Characterization

**Figure 3:**
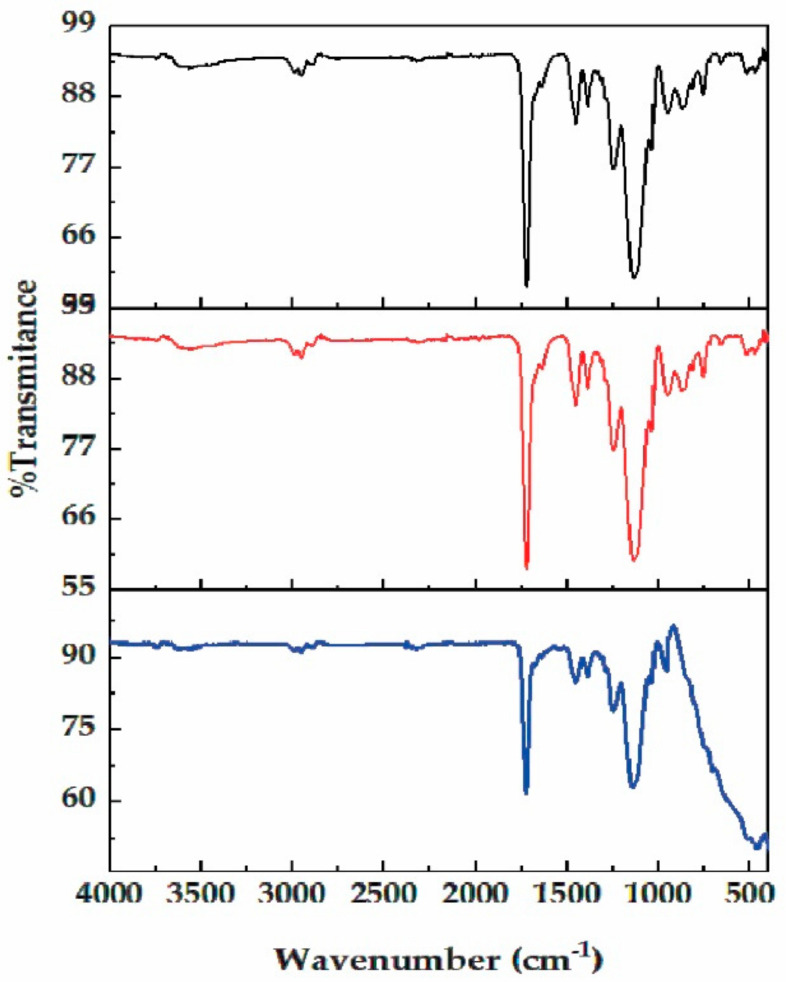
FT-IR spectra of the MIP, IIP, and NIIP (black, red, and blue lines, respectively).

Considering the functionality of the copper complex to build the polymeric structures, the vinyl polymerizable groups of VBA ligands and the carboxylic coordination sites could be pending functionalities attached to the polymeric chain formed with the comonomer EGDMA; in these cases, cross-linked materials are usually obtained, some examples of which were previously reported [12,19,21,23]. A second reaction site in the copper complex could be the saturated coordination site with a water molecule that can be released by exchange reaction with ligands showing an increased binding affinity. Then, the MIP was prepared by the free radical polymerization reaction, using the copper complex as the functional monomer, EGDMA as the cross-linker comonomer, DMF as the porogen solvent, and the AIBN as the initiator. Scheme 1 shows the general steps of the preparations of the imprinted materials. The temperature of the reaction was maintained at 70 °C to guarantee the thermal decomposition of AIBN as reported by this azo initiator [27]. The MIP was obtained as a green solid with crystalline morphology. The polymerization methods varied: emulsion, mass, and coprecipitation synthesis, where the choice of method depends on the morphology, as well as the physicochemical characteristics desired in the polymer. In emulsion polymerization, a hydrophobic organic porogen (in which the polymerization mixture was found) and an organic or aqueous dispersing medium were combined, which were immiscible and formed two phases. This type of polymerization occurred in the drops of the polymerization mixture, with agitation and constant temperature. The mass polymerization method consisted of the synthesis of the polymer in the porogen; this method did not require agitation and was performed merely by adding the initiator to the pre-polymerization mixture at a constant temperature. One of the main features in these two methods was the choice of the porogen as well as its solubility in the monomer, which resulted in different ordering variants. In this experiment, we used two methods, mainly changing the porogen. The porogens used were dimethylformamide and nitromethane (see Supplementary Material) [12,28]. The IIP was obtained as a pale-yellow crystalline solid after removing the ion copper(II) template by an acid wash of the crystalline MIP, as shown in Scheme 1. The functional monomer can be dissolved in methanol, DMF, and DMSO. However, the [Cuphen(VBA)_2_H_2_O-*co*-EGDMA]*_n_* showed poor solubility properties in solvents such as water, ethanol, methanol, acetonitrile, DMF, DMSO, and THF. The IIP and the NIIP had the same properties as the MIP in the solvents mentioned. The characteristic low solubility in organic solvents of the MIP, IIP, and NIIP prepared here could be related to the presence of a high number of double bonds resulting in cross-linking units for the resulting polymer structure [29]. To obtain chemical purity of the materials, the unreacted monomer, ligands, and copper salt were washed with water and methanol. All the prepared imprinted and non-imprinted polymers were obtained with an adequate grade of purity because of their crystallinity. It is clear from the inset photos of Scheme 1 that the color of these materials was different. The color was characteristically blue for the functional monomer with the presence of copper ion in a square pyramid coordination complex [24]. Then, the color turned green in the case of MIP, in which the addition of EGDMA to the chain and the loss of a water coordination molecule probably induced a distortion of the geometry on the coordination sphere. The pale yellow and white colors of the ion-imprinted and non-imprinted polymers could be interpreted by the presence of only the organic ligands. However, in the case of IIP, the cross-linked structure in which ligands were preorganized by the template resulted in a difference compared with the non-organized structure of NIIP. The infrared spectra of MIP, IIP, and NIP from Figure 3 helped support the comments made above. In general, it was found that the FT-IR spectra of the polymers prepared were very similar because all the materials were synthesized with the same methodology and precursors [23,30]. However, in the case of IIP, the preorganized structure was conserved, and the corresponding imprinted cavities were available for ion adsorption application. The FT-IR spectra of the prepared materials shared some expected signals around 1100–1200 cm^−1^, and 1721 cm^−1^ due to the C=O from the EGDMA. One of the infrared signals that verified the polymerization process was the intensity change of the C=C signal in comparison to the medium intensity signal at 1546 cm^−1^ assigned to the vinyl group from the VBA ligands that appeared in the functional monomer spectra of Figure S1 [19,30]. Additionally, the tert-butyl, a structure that is formed when starting the polymerization chain, was observed at 1250–1260 cm^−1^. It is important to notice from Figure 3 that no additional signals from free ligands have appeared in the spectra.

On the other hand, the functional monomer mid-infrared spectrum (see Figure S1, Supplementary Materials) showed the typical signals of the ligands attached to the template copper ion through the N and O atoms. A strong and sharp signal at 1585 cm^−1^ was assigned to the asymmetric vibrational mode of the O—C—O group. At 1370 cm^−1^, a strong and sharp signal corresponded to the symmetric vibrational mode of the O—C—O group. The medium-intensity C—H signals were found around 1150 cm^−1^. Two short and sharp signals were found at 838 and 720 cm^−1^ due to the N=C of the phenanthroline ligand and the short signals around 772 cm^−1^ for the aromatic groups. The corresponding Δν_as-sym_(COO) for the monomer was 215 cm^−1^. These values agree well with the monodentate coordination mode of the carboxylic groups from the VBA ligands [31]. The broad and strong signal around 3454 cm^−1^ was assigned to the O—H stretching vibrations of lattice water molecules in the functional monomer [24], which suffered a notable intensity diminished in the spectra from Figure 3, possibly attributed to the loss of water molecules during the polymerization process.

### 3.3. Surface Area Properties

The results obtained for the surface area properties of the imprinted polymers indicated that the Brunauer-Emmett-Teller (BET) surface area (S_BET_) was 0.3496 and 0.2549 m^2^/g for MIP and IIP, respectively. The total pore volumes (*V_T_*) were 0.000384 and 0.000008 cm^3^/g, and the corresponding average pore diameters (*D_p_*) were 4.3986 and 0.1180 nm. The relative decrease in S_BET_ in the case of IIP could be precisely attributed to the satisfactory removal of the copper ion template after the MIP leaching process. In addition, the *V_T_* of IIP presented a decrease in comparison to the *V_T_* of MIP because, along the active sites of these adsorbents, the formulated molecular complexes were different, thus showing a specific development of porosity [4,32]. In agreement with the IUPAC definition, the MIP could be considered as a mesoporous material because its pore diameter is around 2–50 nm, and the IIP could be a microporous material because its pore diameter is less than 2 nm [4]. It is very desirable to obtain microporous materials as in this case because the affinity for ion remotion is improved. Copper ion has an ionic radius of 0.087 nm, closer to the IIP pore diameter because the pore of IIP is precisely where templated by the lixiviation of the Cu^2+^. Then, a high selectivity and chemical functionality of the imprinted polymer could be expected. The specific surface area of NIIP was determined by the relatively easy and inexpensive method of methylene blue adsorption. Then, the specific surface area was defined as the accessible area of the solid surface per unit mass of material [19,20,33]. For comparison, the IIP surface was also determined by this method. The results showed a surface area of IIP higher than NIIP, being 12.60 and 11.30 m^2^/g, respectively. By leaching the Cu(II) ions from the polymer matrix, we obtain the formation of specific cavities in the polymer network, resulting in a higher surface area in the IIP than in the NIIP [34].

**Figure 4:**
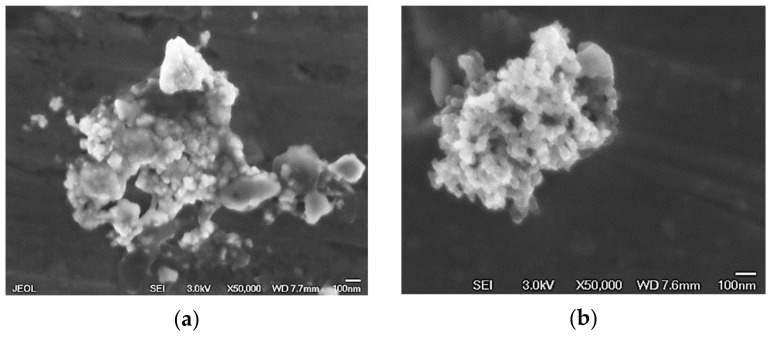
SEM micrographs of the imprinted polymers, (**a**) image at 50,000× of MIP and (**b**) image at 50,000× of IIP.

To improve the adsorption of the superficial area of the imprinted polymers, a porous structure is desirable in which the binding sites at the surface are exposed. In Figure 4, the scanning electron micrographs of MIP and IIP show that their surface is morphological with the presence of meso, as well as micropores across the surface due to the influence of the cross-linking, and the imprinting reaction [4]. This morphological feature of imprinted polymers was also in agreement with the BET results mentioned above. Figure 4a shows a material with irregular particles tending to a smoothly prismatic and spherical surface with an average diameter of 90.73 nm, corresponding to the molecular imprinted polymer. Figure 4b exhibits monoliths of a material with regular particles packed together and a smooth spherical surface with an average diameter of 41.33 nm, corresponding to the ion-imprinted polymer. As shown in Figure 4 and Table S1 (Supplementary Materials), the order of increase of the particle diameter of the prepared materials was IIP < MIP < functional monomer. The presence of many micropores on the spherical surface of the IIP is more beneficial to the fast and homogeneous binding of template ions.

### 3.4. Static Adsorption Capacity and Adsorption Isotherm

**Figure 5:**
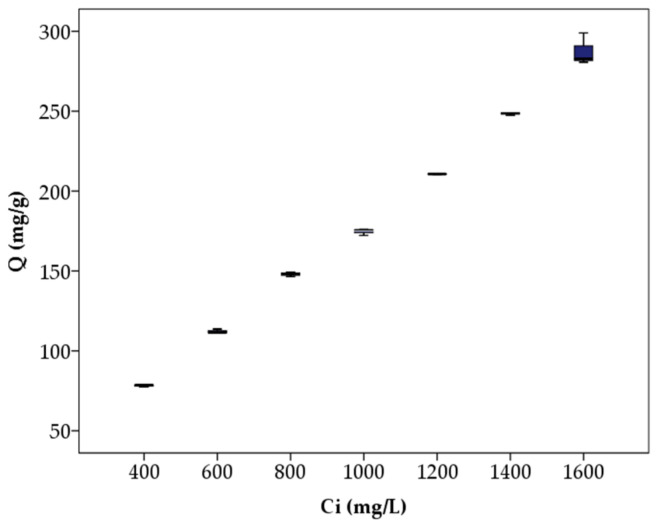
Effect of initial concentration of Cu(II) on adsorption capacity of ion-imprinted polymer.

The maximal concentration of metal ions adsorbed by the ion-imprinted polymer at equilibrium is stated by the adsorption capacity [21,22]. The amount of copper ions adsorbed per unit mass of IIP, almost to saturation, against the initial concentration of Cu(II) is shown in Figure 5. Even though the range of concentrations used in this study was higher than in other studies, a plateau trend was not reached [21,35].

The maximum static adsorption capacity of the IIP was 287.45 mg/g, being higher with respect to other copper ion-imprinted polymers described in previous publications, with adsorption capacity in the range of 16.55 and 132.77 mg/g [11,12]. A list of maximum adsorption capacities for copper with various adsorbents is shown in Table 3. The adsorbent based on IIP structures has higher sorption capacities for copper than the other materials. The imprinted technology allows us to obtain materials like the MIP and IIP presented in this work, which are highly sensitive materials, with high selectivity and affinity for the analyte, because they have selective molecular recognition properties.

**Figure 6:**
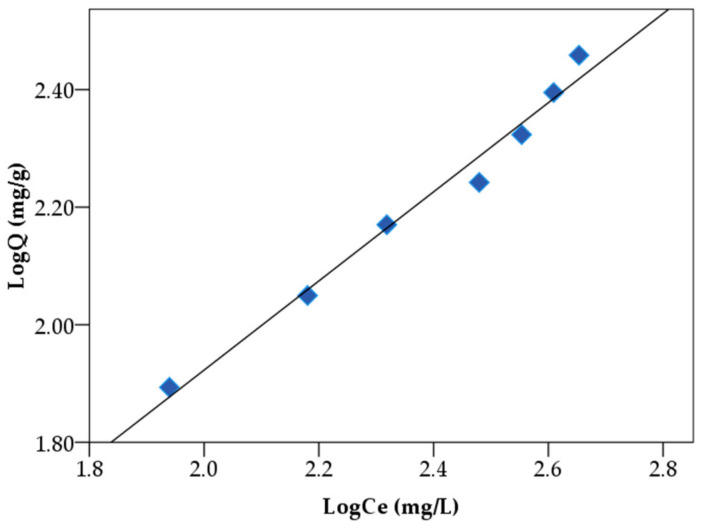
Freundlich plot of copper ions on ion-imprinted polymer. Linear equation *y* = 0.758*x* + 0.407, *R*^2^ = 0.981.

Heavy metals or adsorbates can interact with the available binding surfaces of the ion-imprinted polymers through an adsorption process. The study of adsorption isotherm is needed to interpret the mechanism of adsorption between IIP and adsorbates and to standardize its application. The adsorption isotherm describes the relationship between the equilibrium of any solute in the solution and the adsorbent. According to the shape of the adsorption curve, the possibilities of the adsorption process occurring can be defined. The Langmuir and Freundlich isotherms are commonly used in water treatment [7,8]. The adsorption isotherm from Figure 6 presents a type of linear adsorption with *y =* 0.758*x* + 0.407 and *R*^2^ = 0.981, where the mass of the solute in the aqueous solution and the mass of solute adsorbed on the solid matrix are kept in equilibrium. There are certain conditions that favor the existence of a linear isotherm; among them, the most important ones are as follows: existence of flexible molecules in the medium due to different degrees of crystallization of the material and a greater affinity of the solute with the substrate than with the solvent. Therefore, the Freundlich model fit better than the Langmuir model when the IIP was employed to remove Cu^2+^, with a high correlation coefficient compared to the Langmuir model (*R*^2^ = 0.686). Due to the Freundlich model’s improved fit, it can be deduced that the adsorption of heavy metal ions onto the IIP heterogeneous surface is classified as a multilayer.

### 3.5. Selective Recognition

**Scheme 2:**
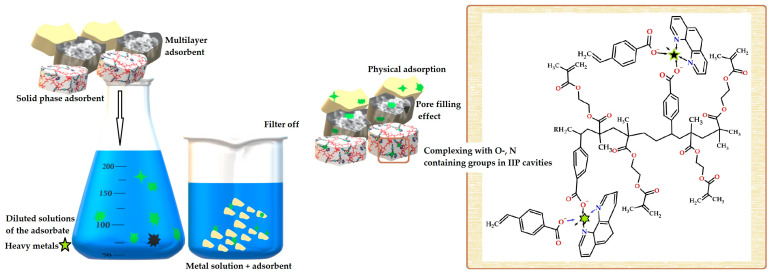
Adsorption mechanisms of heavy metals by the ion-imprinted polymer.

The selective recognition of IIP by Cu(II) against Ni(II) from their solutions was studied. Nickel ion was selected as a competitor sorption of copper, given that the metal ions of the same charge and similar ionic radii as Cu(II) can influence the adsorption process of copper ions. Then, copper and nickel have the same charge of 2+, and the ionic radii are very close, 0.087 for copper and 0.083 nm for nickel. The imprinting effect generated by the template used in the imprinted polymer could be quantified with the selectivity coefficient determination. The results of our experiments indicated a distribution coefficient of 950 and 590 mL/g corresponding to Cu(II) and Ni(II); this is interpreted as the Cu-IIP complex stability being higher than the Ni-IIP complex. Then, the IIP has a good imprinting effect, with a selectivity coefficient of 1.61 [19]. Scheme 2 describes the mechanism of heavy metal adsorption using the ion-imprinted polymer as a multilayer array in agreement with the Freundlich model. In the physical adsorption, the adsorbates are adhered to the available binding surfaces of IIP, filling the micropores of the material, and metal ions can interact with the polymeric matrix through coordination bonds.

## 4. Conclusions

In this work, a Cu(II) ion-imprinted polymer was prepared and characterized. First, the functional monomer of [Cuphen(VBA)_2_H_2_O] was synthesized. Then, the molecular imprinted polymer of [Cuphen(VBA)_2_H_2_O-*co*-EGDMA]*_n_* was obtained by free radical polymerization. The copper complex, ethylene glycol dimethacrylate, and 2,2-azobisisobuthyronitrile were used as the functional and cross-linking monomers and the initiator, respectively. The Cu(II) ions were leached in an acidic wash, and the IIP was obtained. The polymers were characterized by some physical and spectroscopical techniques. The morphology of the imprinted materials showed some irregular surface particles for MIP, and the IIP surface area consisted of packed, smoothly spherical particles. Moreover, the MIP is a mesoporous material with a pore diameter of around 4 nm. The IIP with a pore diameter of less than 2 nm was considered as microporous material. The maximum adsorption capacity of IIP was 287.45 mg/g, and this behavior was represented by the Freundlich adsorption isotherm linear model. The Ni(II) was used as the competitive specie against Cu(II); then, the selective recognition of IIP was 1.61 L/g. These results showed that IIP has a high potential for being used for copper or similar chemical species in contaminated water adsorption. In conclusion, we focused on the synthesis of IIP whose preorganized structure came from a metal complex used as a functional monomer. Thus, the relatively facile preparation, cross-linked structure, and low solubility in water rendered the IIP a promising sorbent for selective extraction of metal ion applications in water pollution with specific characteristics like Cu(II) based in an ionic radius, electronegativity, electronic configuration, and geometry. Then, further studies on its selectivity in the presence of other metal ion competitors such as Co(II), Cd(II), Zn(II), Pb(II) would be fundamental. The mechanism of the contaminant removal procedure, regeneration of the material, as well as proceeding with reuse cycles of this IIP are some of the next experiments for future research.

## Figures and Tables

**Table 1:** Crystal data and structure refinement for the functional monomer [Cuphen(VBA)_2_H_2_O].

Empirical Formula	C_30_H_24_CuN_2_O_5_
Formula weight	556.05
Temperature	130(2) K
Wavelength	0.71073 Å
Crystal system	Triclinic
Space group	P-1
Unit cell dimensions	a = 8.0188(4) Å
	b = 10.7541(10) Å
	c = 15.7861(14) Å
	α = 101.821(7)°
	β = 94.259(6)°
	γ = 109.056(7)°
Volume	1244.68(18) Å^3^
Z	2
Density (calculated)	1.484 g/cm^3^
Absorption coefficient	0.923 mm^−1^
F(000)	574
Crystal size	0.540 × 0.380 × 0.240 mm^3^
Theta range for data collection	3.751 to 26.055°
Index ranges	−9 <= h <= 9, −13 <= k <= 10, −19 <= l <= 19
Reflections collected	8643
Independent reflections	4908 [R(int) = 0.0293]
Completeness to theta = 25.242°	99.7%
Refinement method	Full-matrix least-squares on F^2^
Data/restraints/parameters	4908/5/350
Goodness-of-fit on F2	1.056
Final R indices [I > 2sigma(I)]	R1 = 0.0412, wR2 = 0.0950
R indices (all data)	R1 = 0.0528, wR2 = 0.1029
Largest diff. peak and hole	0.483 and −0.556 e.Å^−3^

**Table 2:** Selected bond lengths [Å] and angles [°] for the functional monomer [Cuphen(VBA)_2_H_2_O].

Bond Lengths (Å)		Bond Angles (°)	
C(1)—N(1)	1.323(3)	O(3)—Cu(1)—O(1W)	94.99(8)
C(1)—C(2)	1.402(4)	O(3)—Cu(1)—N(2)	89.33(8)
C(13)—O(1)	1.258(3)	O(1W)—Cu(1)—N(2)	165.47(8)
C(13)—O(2)	1.266(3)	O(3)—Cu(1)—N(1)	166.89(8)
C(29)—C(30)	1.301(4)	O(1W)—Cu(1)—N(1)	91.43(8)
Cu(1)—O(3)	1.9493(18)	N(2)—Cu(1)—N(1)	81.77(8)
Cu(1)—O(1W)	1.9823(19)	O(3)—Cu(1)—O(1)	99.29(7)
Cu(1)—N(2)	2.007(2)	O(1W)—Cu(1)—O(1)	91.17(7)
Cu(1)—N(1)	2.026(2)	N(2)—Cu(1)—O(1)	101.83(7)
Cu(1)—O(1)	2.2622(17)	N(1)—Cu(1)—O(1)	91.97(7)

**Table 3:** Comparison of maximum adsorption capacity of copper(II) ions using different adsorbents.

Adsorbents	Maximum Adsorption Capacity (mg/g)	References
Granular activated carbon	48.22	[8]
Geopolymers	35.88–152.3	[7]
Activated carbon	75.0	[35]
Ion-imprinted polymer	287.45	This work

## Data Availability

Data is available from the corresponding author on reasonable request.

## Supplementary Materials

The following supporting information can be downloaded at: https://www.mdpi.com/article/10.3390/polym15051186/s1, Figure S1: FT-IR spectra of the functional monomer; Table S1: SEM micrographs and average particle diameters of the prepared materials, IIP, MIP and functional monomer.
