# Supplementary material for: Ion-Imprinted Polymer Structurally Preorganized Using a Phenanthroline-Divinylbenzoate Complex with the Cu(II) Ion as Template and Some Adsorption Results
Source: Polymers (Basel). 2023 Feb 26;15(5):1186. doi: 10.3390/polym15051186 (PMC10007393; doi:10.3390/polym15051186)
Supplement: Supplementary file 1 [file polymers-15-01186-s001.zip › polymers-2095637-SM.pdf]

*Supplementary Materials*

# Ion-Imprinted Polymer Structurally Preorganized Using a Phenanthroline-Divinylbenzoate Complex with the Cu(II) Ion as Template and Some Adsorption Results


**Egla Yareth Bivián-Castro [1,*], Abraham Zepeda-Navarro [1], Jorge Luís Guzmán-Mar [2], Marcos Flores-Alamo [3] and Brenda Mata-Ortega [1].**

[1] Centro Universitario de los Lagos, Universidad de Guadalajara Av. Enrique Díaz de León 1144, Col. Paseos de la Montaña, Lagos de Moreno 47460, Jalisco, Mexico
[2] Facultad de Ciencias Químicas, Universidad Autónoma de Nuevo León (UANL), Ave. Universidad s/n, Cd. Universitaria, San Nicolás de los Garza, 66455, Nuevo León, Mexico
[3] Facultad de Química, Universidad Nacional Autónoma de México, Ciudad Universitaria, Ciudad de México 04510, Ciudad de México, Mexico

* Correspondence: egla.bivian@academicos.udg.mx; Tel.: +52(474)7424314 ext. 66576


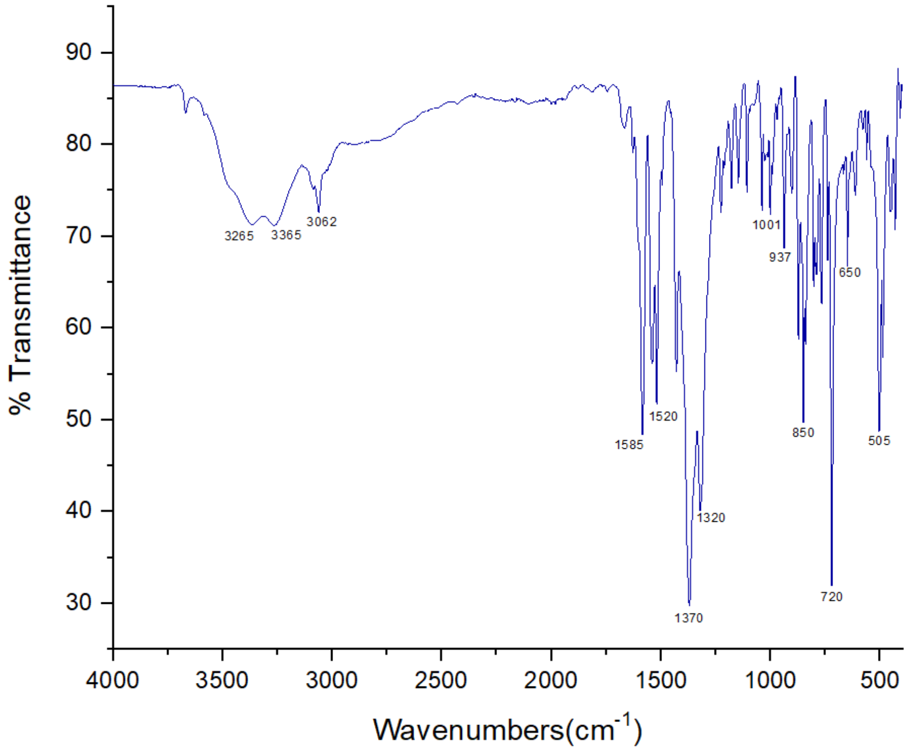


**Figure S1.** FT-IR spectra of the functional monomer.

**Preparation of the MIP with Nitromethane as the Porogen.**

In 10 mL of nitromethane was dissolved 0.18 mmol (0.1 g) of the copper complex, [Cuphen(VBA)2H2O]. Then were added 3.59 mmol (678 µL) of EGDMA and 0.02 mmol (0.0033 g) of AIBN previously recrystallized in methanol. The reaction was left under constant stirring for 48 h at 70 oC and under inert atmosphere conditions. A brownish-green precipitate was filter off, washed and dry under vacuum. The resulted blue precipitated was filter off then it was dissolved in 15 mL of methanol and left under fridge to obtain crystals available for X-ray diffraction.

**Table S1. SEM micrographs and average particle diameters of the prepared materials, IIP, MIP and functional monomer.**

| | |
|---|---|
| Monomer | |
| 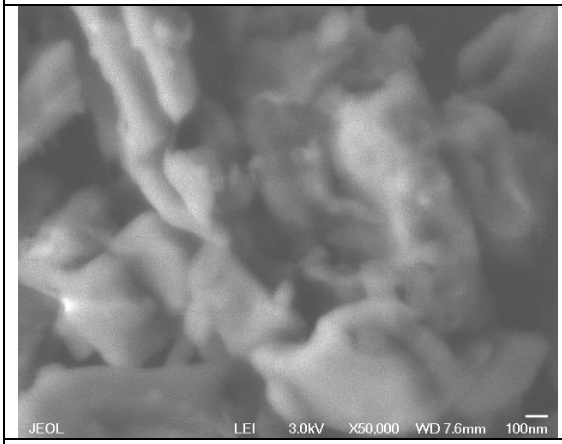  | Average diameter of the particles is 101.59 nm |
| MIP | |
| 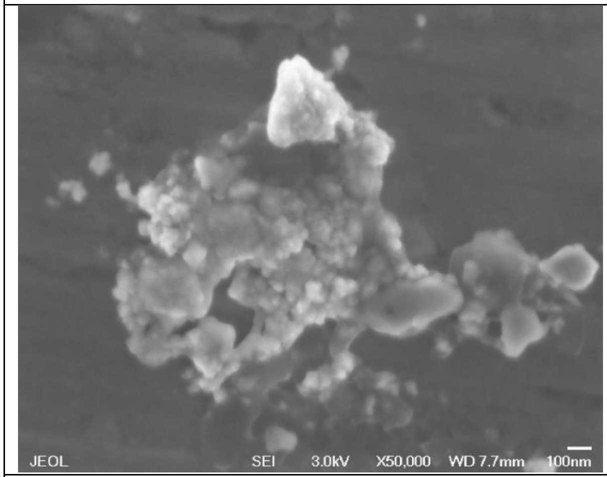  | Average diameter of the particles is 90.73 nm |
| IIP | |
| 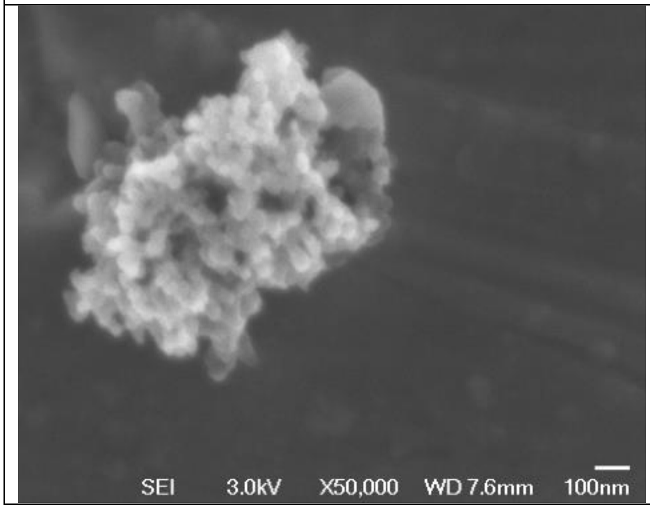  | Average diameter of the particles is 41.33 nm |
